# Cpf1 nucleases demonstrate robust activity to induce DNA modification by exploiting homology directed repair pathways in mammalian cells

**DOI:** 10.1186/s13062-016-0147-0

**Published:** 2016-09-14

**Authors:** Eszter Tóth, Nóra Weinhardt, Petra Bencsura, Krisztina Huszár, Péter I. Kulcsár, András Tálas, Elfrieda Fodor, Ervin Welker

**Affiliations:** 1 Institute of Enzymology, Research Centre for Natural Sciences of the Hungarian Academy of Sciences, 2 Magyar Tudósok krt., Budapest, H-1117 Hungary; 2 Institute of Biochemistry, Biological Research Centre, Hungarian Academy of Sciences, Szeged, H-6726 Hungary; 3 University of Szeged, Szeged, H-6726 Hungary; 4 Semmelweis University, Budapest, H-1085 Hungary

**Keywords:** Cpf1 nuclease, AsCpf1, LbCpf1, CRISPR, Cas9 nuclease, GFxFP assay, SpCas9, SaCas9, NmCas9, StCas9, Genome engineering, RNA, DNA

## Abstract

**Background:**

Cpf1 nucleases have recently been repurposed for site-specific genome modification. Two members of the Cpf1 family, the AsCpf1 from *Acidaminococcus sp.* and the LbCpf1 from *Lachnospiraceae* bacterium were shown to induce higher indel frequencies than SpCas9 when examining four randomly-selected target sequences for each type of nuclease. Whether they are a real match for Cas9 nucleases, however, remains to be verified.

**Results:**

Here, we used AsCpf1 and LbCpf1 to induce homology directed repair, either single strand annealing (SSA) or homologous recombination (HR), in N2a mouse neuroblastoma cells. Exploiting a plasmid that contains two GFP halves with overlapping sequences and exploring 20 targets, on all but one both nucleases consistently performed with above 10 % efficiency. Several Cas9 nucleases have been previously characterised in order to find an orthogonal counterpart for the most widely used promiscuous SpCas9. Here, we found that AsCpf1 and LbCpf1 might be better candidates than three of the best such counterparts: Cas9 from *Staphylococcus aureus*, from *Streptococcus thermophilus* and from *Neisseria meningitidis*, when assessed for inducing efficient SSA mediated repair in N2a cells. When tested on genomic targets exploiting HR, both nucleases were able to induce the integration of a donor cassette with 1000 bp-long homologous arms. We also generated plasmids that express these Cpf1 nucleases together with their cognate crRNAs and that are equipped with type IIS restriction enzyme sites to facilitate spacer cloning.

**Conclusions:**

Our results suggest that employing As- or LbCpf1 nuclease to induce homology directed repair in N2a cells, although is less effective at present than employing SpCas9, it is an equally or more effective tool than the most frequently used orthogonal Cas9 counterparts of SpCas9. These findings support the position of Cpf1 nucleases on the side of SpCas9 on the palette of effective genome engineering tools.

**Reviewers:**

This article was reviewed by Eugene Koonin, Haruhiko Siomi and Jean-Yves Masson.

**Electronic supplementary material:**

The online version of this article (doi:10.1186/s13062-016-0147-0) contains supplementary material, which is available to authorized users.

## Background

Although recent studies suggest that clustered regularly interspaced short palindromic repeat (CRISPR)-associated protein (Cas) systems are also used for non-defense roles in the host [1, 2], they are best known as part of the adaptive immune systems of prokaryotes that have recently been exploited for generating extremely powerful genetic devices [3–6]. The CRISPR-Cas system of the host grants specific resistance against phages and plasmids by keeping “memories” of past infections through acquiring short DNA fragments and destroying the reinvading agents that are identified based on these short, 20-30 nucleotide-long sequences [7, 8]. The adaptation modules that acquire and deposit appropriate sequence motifs from the invading agent to specific locations called CRISPR arrays of the microbial genome, consist of two proteins: Cas1 and Cas2 [9]. The effector complexes show much greater diversity in terms of the participating Cas proteins, across various prokaryotic CRISPR-Cas systems, and are being divided into two classes: Class 1 systems that utilize multiple subunit effector complexes, and Class 2 systems that work with a single large effector Cas protein [10]. Within the two classes five types of complexes are being distinguished (type I, III and IV of Class 1 and type II and V of Class 2) according to the presence of one of the signature proteins: Cas3, Cas10, Csf1, Cas9, and Cpf1, respectively [11–13]. Cas9 proteins, effectors of the type II Class 2 systems have attracted particular attention in the past few years due to their possible harnessing for genome editing that quickly revolutionized the way we are capable to execute site-specific modification of complex vertebrate genomes today [3–5, 13, 14]. Cas9 proteins are also exploited for the purpose to regulate transcription and epigenetic states at specific genomic locations [15–22]. The power of Cas9 as a genome manipulating tool lays in its easy programming. Altering the so called spacer sequence of its associated RNA molecule – that determines the specificity of the nuclease – the Cas9 protein can target different DNA sequences: i.e., those that contain complementary sequences (protospacers) to the chosen spacer sequence and also are immediately followed by a short DNA motif called protospacer adjacent motif (PAM). The PAM is recognized by the protein’s PAM-binding domain and its sequence may differ for Cas9 proteins from different species [22, 23].

Recently, Cpf1 (CRISPR from *Prevotella* and *Francisella* 1) nucleases – single effector proteins of the type V Class 2 systems – have been investigated for their possible applicability for genome engineering tasks. Zetsche et al. demonstrated that the Cpf1-containing CRISPR-Cas locus of *Francisella novicida U112* encodes a functional defense system that is capable of mediating plasmid interference in bacterial cells [24]. This work revealed three features of Cpf1 of *Francisella novicida* (FnCpf1) that seem to be general among Cpf1 nucleases but are distinct from those of Cas9s. First, FnCpf1 works with a single CRISPR RNA (crRNA) in contrast to Cas9 that also requires a trans-activating CRISPR RNA (tracrRNA) for crRNA processing and target recognition activity. Second, the PAM of FnCpf1 is rather T-rich in contrast to the more G-rich Cas9 PAMs. Third, while Cas9 nucleases produce blunt-end termini at double strand breaks, FnCpf1 produces 4- or 5-nt-long 5’ overhangs [24]. Initially, Cpf1 nucleases were proposed to act as dimers based on the fact that they contain only one known nuclease domain (RuvC) and the inactivation of this domain leads to a completely inactive Cpf1 in contrast to Cas9 where the inactivation of one nuclease domain generates a nickase [24]. However, it is not clear how the concerted action of two, dimerized Cpf1 proteins might result in a double strand break. Recent studies examining the crystal structure of one of the Cpf1 nucleases, the *Lachnospiraceae bacterium ND2006* Cpf1 (LbCpf1), and carrying out biophysical assays on FnCpf1 showed that they display no oligomerisation upon binding to crRNS and/or DNA [25, 26]. The crystal structure of *Acidaminococcus sp. BV3L6* Cpf1 (AsCpf1) reveals a nuclease domain with a new fold responsible for the cleavage of the target strand. Mutation of the conserved residues of this domain abolishes the double strand breaking activity of the nuclease, leading to a nickase [27]. These distinct features of Cpf1 nucleases might render them a very useful alternative genome manipulating tool to Cas9s. This contention was further strengthened by recent studies on the activity of Cpf1s on off-target sequences [28–30]. Genome-wide off-target analyses and targeted deep sequencing suggest that Cpf1 nucleases tolerate only one or two mismatches in contrast to SpCas9 that has been reported to tolerate 5–6 mismatches [28–30].

Zetsche et al. examined 16 members of the Cpf1 nuclease family. Two of them AsCpf1 and LbCpf1 were proved to mediate efficient genome editing in HEK293FT cells. In these experiments, exploring a limited set of four targets and non-homologous end joining (NHEJ) repair, the indel frequencies caused by AsCpf1 and LbCpf1 were actually higher than that of SpCas9 [24].

Here, we investigated the activity of AsCpf1 and LbCpf1 nucleases on a number of various targets in N2a mouse neuroblastoma cells exploiting two homology directed repair (HDR) pathways: single strand annealing (SSA) and homologous recombination (HR), in contrast to NHEJ repair, and compared their activity to various Cas9 proteins.

## Results

In order to monitor the activity of AsCpf1 and LbCpf1 nucleases we employed a fluorescence reporter assay using an interrupted-GFP expression cassette in a plasmid where the two fragments (GFP “halves”) of the GFP sequence are separated in a way to contain an overlapping 480 base-pairs long region (Additional file 1) [31]. The target site for the nuclease is placed between the two halves and upon nuclease cleavage the generated double strand DNA break is repaired by single strand annealing (SSA) directed by the overlapping homologous sequences (Additional file 1). A presumably small fraction of the breaks might also be repaired by homologous recombination between two plasmids. The advantage of this assay, that we refer to as “GFxFP assay”, is that it allows measuring the ability of a nuclease to mediate HDR by cleaving a specific target while the cleavage efficiency is not influenced by factors like the epigenetic state of the targeted locus or the relative distance of the target from the homologous arms.

In order to test this assay system we used SpCas9 with two targets that had been tested earlier in either mammalian (PrP10) or bacterial (Sp1) cells (unpublished results). The interrupted GFP plasmids containing either the Sp1 or the PrP10 target (see Additional file 2: Table S1 for the target sequences) were cotransfected into N2a mouse neuroblastoma cells with a vector expressing SpCas9, the corresponding guideRNA (gRNA) and the fluorescent protein iRFP670 [32]. The latter facilitated monitoring of transfection efficiency. As control, a vector expressing an inactive SpCas9 (dead, dSpCas9) was used for transfecting the cells. The number of fluorescent cells was counted two days after transfection. The Sp1 target resulted in 73 %, while the PrP10 target resulted in 54 % fluorescent cells in the transfected populations, respectively (Additional file 1). Surprisingly, the control population also showed a remarkable percentage, about 30 % GFP positive cells (Additional file 1 – EGxxFP). Apparently, in a fraction of the plasmids recombination between the homologous sequences occurred without nuclease cleavage.

**Fig. 1:**
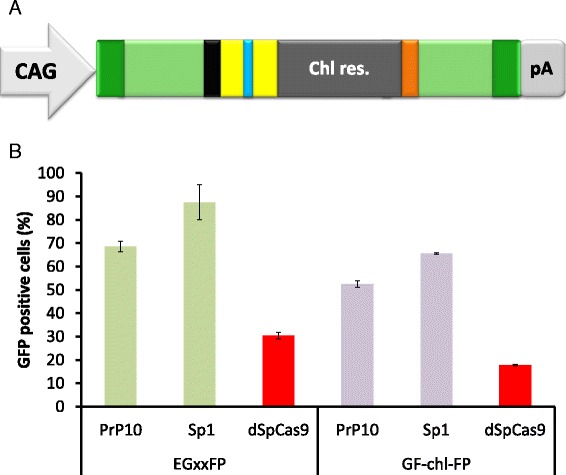
Insertion of a chloramphenicol resistance gene decreases the number of fluorescent cells measured in a GFxFP assay. **a** Schematic representation of the interrupted GFP cassette, depicted as on Additional file 1, with a chloramphenicol resistance gene (*middle dark grey* box) inserted between the homologous sequences of the GFP halves **b** The percentages of GFP positive cells are compared when using the reporter assay with either the original (EGxxFP, *green* bars) or the additional chloramphenicol resistance gene containing (GF-chl-FP, *purple* bars) plasmids with the interrupted GFP-halves. In each case, two targets were tested, PrP10 and Sp1, using SpCas9 nuclease and the corresponding gRNAs along with the reporter plasmids in N2a cells. As controls, inactive SpCas9 was used in both cases (*red* bars, dSpCas9). Values are normalized to the transfection efficiencies measured by using the fluorescence of iRFP670 used as transfection control. Error bars show the mean ± standard deviation of percentages measured in *N* = 3 independent transfections. The insertion of the chloramphenicol cassette decreased the number of the fluorescent cells in both the active- and dead SpCas9 containing samples

Next, to account for the possibility of recombination events that may already take place in the bacterial cells while handling the plasmid, either a chloramphenicol resistance gene (pGF-chl-FP) or the plasmid replication of origin (pGF-ori-FP) was placed within the region between the homologous sequences of the GFP halves (Fig. 1a). After plasmid isolation from bacterial cells we found no indication of a recombined fraction occurring in either of the cases (pGF-ori-FP and pGF-chl-FP plasmids) when probed on agarose gel after restriction enzyme digestions (data not shown). This suggests that such a population, if exists, is less than 1 %. We cloned the previously used Sp1 and PrP10 targets into these vectors as well, and tested the SpCas9-induced repair of GFP in N2a neuroblastoma cells. The insertion of the chloramphenicol cassette resulted a decrease in the number of the fluorescent cells in the active SpCas9 containing samples (Fig. 1b). When the inactive dSpCas9-expressing vector was cotransfected with the pGF-chl-FP plasmid, GFP positive cells could still be detected, although at a lower percentage than in the case of the unmodified original plasmid, EGxxFP (Fig. 1b). This suggests that functional GFP molecules were still expressed in a fraction of the pGF-chl-FP modified plasmids without nuclease cleavage. The observed decrease in the GFP signal may not only be attributable to a reduction in the fraction of already recombined plasmids in the bacterial cells, but might also reflect the reduced recombination capability of the two GFP halves as the distance between them is increased by the presence of the chloramphenicol resistance cassette. The origin of this relatively high background GFP fluorescence is not fully understood (Additional file 3), but it may partially come from a somewhat high level of naturally occurring double strand breaks present in N2a cells. When comparing, anti-H2AX staining revealed that N2a and HEK293T cells had rather comparable but remarkably higher levels of DNA double strand breaks than HeLa cells (Additional file 3).

Since we observed that the detected fluorescence varied from experiment to experiment (possibly reflecting the actual condition of the culture) but remained relatively stable within the parallel transfections, here and in further experiments we handled the samples to be compared in parallel. Also, we subtracted the corresponding background fluorescence from each sample. We found similar results with the pGF-ori-FP plasmid (data not shown) and hereafter used this plasmid in further experiments.

### AsCpf1 and LbCpf1 nucleases are more efficient when the crRNAs are expressed from a plasmid rather than a PCR-based template

**Fig. 2:**
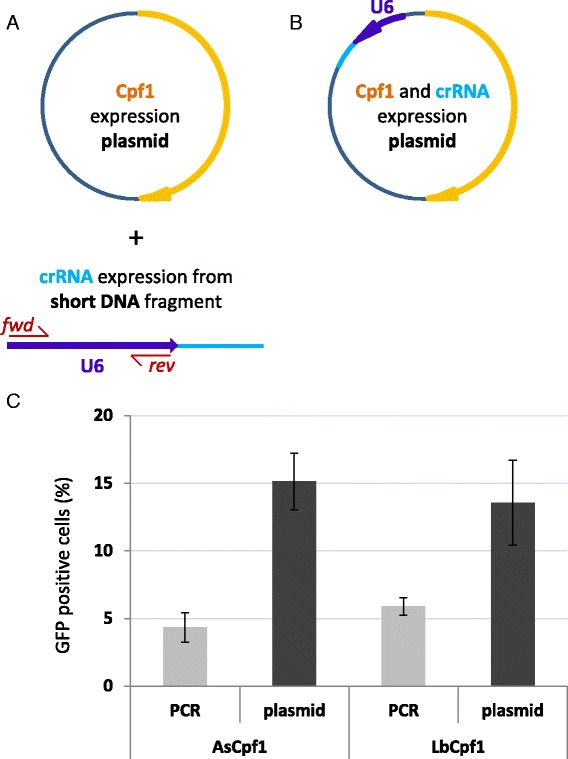
Comparison of efficiencies of the Cpf1 nucleases when used with either a PCR- or a plasmid-derived crRNA in N2a mouse neuroblastoma cells. **a** Scheme of a plasmid template for nuclease expression (*orange*: Cpf1 expression cassette) and of a PCR-based template for crRNA expression [a human U6 promoter PCR product (*blue* with scarlet forward and reverse primers) with a hybridized dsDNA oligonucleotide (*light blue*)]. **b** Scheme of a dual expression plasmid template for both the nuclease (*orange*) and the crRNA (*light blue*) with the human U6 promoter (blue arrow). **c** Percentages of GFP positive cells formed above the background level, induced by the nuclease action of either AsCpf1 or LbCpf1. N2a cells are cotransfected with either a PCR-based template for crRNA and a plasmid for Cpf1 nuclease expression (as depicted on Fig. 2a), labelled as “PCR”, or with a dual expression plasmid for both the nuclease and the crRNA (as depicted on Fig. 2b), labelled as “plasmid”, respectively. GFP positive cells are counted two days after transfection by flow cytometry. All samples are also cotransfected by an mCherry expression vector as a transfection reference and the GFP expression is analysed within the mCherry positive population. The transfection efficiencies measured based on the fluorescence of mCherry ranged within 55.9 % ± 10.5 % when the plasmid was used, whereas 45.0 % ± 8.4 %, when the PCR product. As negative control, a crRNA-less, active AsCpf1 nuclease expression vector was used for each target and the obtained levels of GFP fluorescence for these controls are subtracted from the corresponding samples’ values. Three parallel transfections were made for each case. Error bars show the mean ± standard deviation of percentages measured in *N* = 3 independent experiments. Plasmid-expression of the crRNA results in 2- to 4-fold increase in GFP positive cells as compared to a PCR-template expression

To test the activity of AsCpf1 and LbCpf1 nucleases employing the GFxFP assay we used the DNMT1.3 target [described by Zetsche et al. [24]]. Initially, the crRNA expression cassettes were prepared as described earlier [24], by PCR amplification of the human U6 promoter to which a linker coding the crRNA sequence is ligated (Fig. 2a) and the modified PCR products were cotransfected along with the corresponding Cpf1 nuclease expressing vectors (pY010 or pY016 [24], respectively) into N2a cells. As a result, we were able to detect fluorescence above the background level in case of both nucleases; however, this did not seem to be a robust effect (Fig. 2c). Next, hoping to increase the cleavage efficiency of the nucleases, the expression cassettes for both As- and Lb-crRNA – containing the same DNMT1.3 spacer – were cloned to the corresponding vectors for nucleases after a human U6 promoter (Fig. 2b). Here, as well as in later experiments, the transfection efficiency was monitored using a cotransfected mCherry expression plasmid. Figure 2c shows that expressing the crRNAs from plasmid templates results in 2- to 4-fold increase in GFP positive cells as compared to expression from PCR templates. Thus, plasmids harbouring both Cpf1 and crRNA expression cassettes were used in the further experiments.

To facilitate an easier use of these Cpf1 nucleases against various targets, we cloned the As- and Lb-crRNAs driven by a human U6 promoter into the pY010 and pY016 plasmids [24], respectively, in such a way that the 3’ spacer can be precisely inserted between two sites of a type IIS restriction endonuclease (Esp3I) (Additional file 4). The created plasmids (pTE4396 for AsCpf1 and pTE4398 for LbCpf1) are deposited at Addgene (#74041 and #74042, respectively).

### As- and LbCpf1 induce efficient repair of the GFP cassette using homologous sequences

**Fig. 3:**
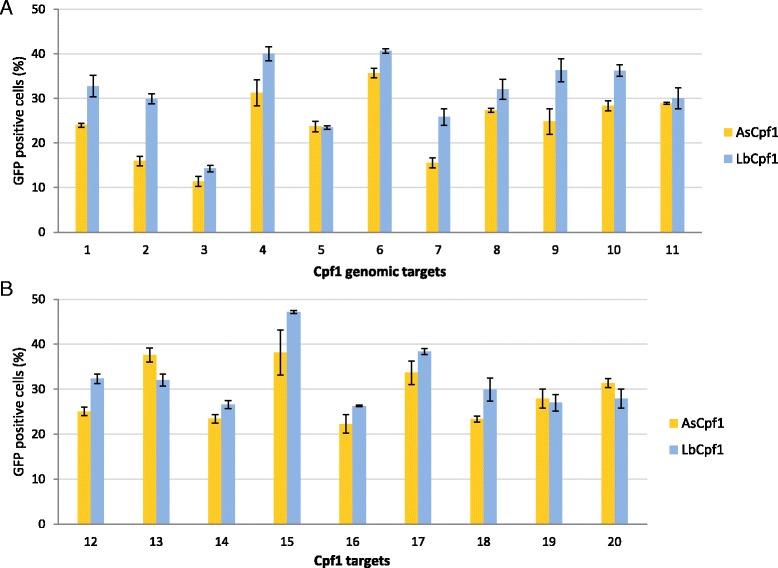
As- and LbCpf1 nucleases can efficiently cleave randomly picked targets. Percentages of GFP positive cells counted above the background level, resulted by the action of various nucleases. Eleven randomly picked targets (**a**) aiming the mouse *PRNP* (1–4), *SPRN* (5) and *PRND* (6–11) genes and further nine arbitrary targets (**b**), cloned into the pGF-ori-FP vector were tested using the GFxFP assay. The target vectors along with the corresponding nuclease vector were transfected into N2a cells and GFP positive cells were counted two days after transfection. All samples are also cotransfected with an mCherry expression vector to monitor the transfection efficiency and the GFP signal is analysed within the mCherry positive population. The background fluorescence was estimated by using a crRNA-less, active AsCpf1 nuclease expression vector as negative control, and the resulting GFP fluorescence found for each target was subtracted from the corresponding sample values. Three parallel transfections were made for each case. Error bars show the mean ± standard deviation of percentages measured in *N* = 3 independent transfections. All of these targets were cleaved efficiently by both nucleases confirming the robustness of the activity of these Cpf1 nucleases

To probe the effectiveness of As- and LbCpf1 nucleases in inducing SSA repair in mammalian cells, 11 mouse genomic targets were selected aiming three mouse prion protein family genes: *PRNP, SPRN* and *PRND* (1-11 in Additional file 2: Table S2). The targets were cloned into the pGF-ori-FP plasmid and the corresponding spacers into the AsCpf1- and LbCpf1-plasmids (pTE4396 and pTE4398, respectively). When tested, both As- and LbCpf1 effectively induced the repair of the GFP cassette with all of the 11 targets, resulting more than 10 % fluorescent cells in all cases (Fig. 3a). We further probed additional nine target sequences (12–20 in Additional file 2: Table S2) by both nucleases. All of these targets were also cleaved efficiently by both nucleases (Fig. 3b), confirming the robustness of the activity of these Cpf1 nucleases.

### Although the utility of As- and LbCpf1 nucleases may be less than that of SpCas9 they do demonstrate comparable efficiencies to three other Cas9 nucleases

**Fig. 4:**
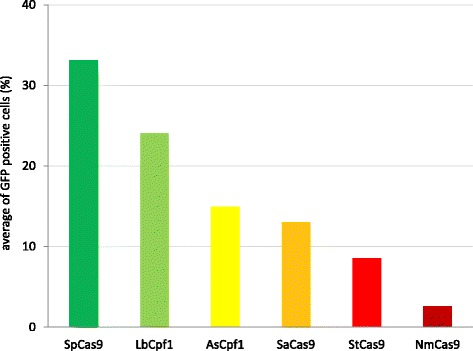
Lb- and AsCpf1 are equally or more efficient to induce SSA than three tested Cas9 counterparts. Percentages of GFP positive cells counted above the background level, resulted by the action of various nucleases. Six nucleases were tested, each of them on five different targets using the GFxFP assay. Columns show the average values obtained for the five different targets in case of each nuclease. GFP positive cells were counted two days after transfection. All samples were also cotransfected with an mCherry expression vector and the results are analysed within the mCherry positive population. GFP fluorescence for negative controls was measured using an inactive SpCas9 expressing vector (Tálas et al., submitted manuscript) and the obtained values are subtracted from each corresponding sample value. Three parallel transfections were made for each case. Average HDR inducing efficiencies were considered by calculating the average of 15 samples. SpCas9: green, LbCpf1: light green, AsCpf1: yellow, SaCas9: orange, StCas9: red, NmCas9: scarlet

Except of the promiscuous SpCas9, Cas9s are generally more selective in their target recognition than the two Cpf1 nucleases used here are. Several Cas9 nucleases have been characterised in order to find an orthogonal counterpart for the most widely used SpCas9 [23]. Here, we picked three of them, the most frequently considered ones: from *Staphylococcus aureus* (SaCas9) [33], from *Streptococcus thermophilus* (StCas9) [23, 34], and from *Neisseria meningitidis* (NmCas9) [23, 35], and compared their robustness to those of AsCpf1 and LbCpf1 using 5-5 arbitrary selected targets for each type of nuclease. The targets were picked randomly, i.e. without employing any prior knowledge of the sequence specificities of the nucleases. This was not the case for SpCas9, where we exploited the existence of gRNA prediction tools: sgRNA designer [36] and sgRNA scorer 1.0 [37] and accepted targets scoring above either 0.5 or 50, respectively. In case of Cpf1 nucleases, representative targets were picked from the 20 targets used on Fig. 3. All targets were tested with the GFxFP reporter system (Additional file 1) that allows a more direct comparison of nuclease activities for inducing SSA homology directed repair, excluding the possible influence of the targets’ relative positions to the homologous arms that is characteristic to HR repair. Interestingly, in this assay both of the Cpf1 nucleases were equally or more effective in inducing HDR on average than the three SpCas9 counterparts: LbCpf1 (24 %) and AsCpf1 (15 %) versus Sa- (13 %), St- (9 %) or Nm- (3 %) Cas9 (Fig. 4 and Additional file 5). As expected, SpCas9 exhibited the most robust activity, although, this might also include a contribution from the prediction tools employed. These results suggest an important position for the Cpf1 nucleases beside SpCas9 on the palette of effective genome engineering tools. When a prediction tool is available to be used for Cpf1 nucleases as well, they might approach even more the utility of SpCas9 in the future.

### Genomic DNA cleavage by As- and LbCpf1 nucleases can induce efficient HDR

In order to check whether the As- and LbCpf1 nucleases can effectively induce homology directed repair on some of the tested targets when these are in their genomic contexts, we exploited a homologous recombination donor molecule (pHRdonor-PrP1000) (Tálas et al., submitted manuscript). The donor molecule contains a promoterless GFP open reading frame (ORF) flanked by 1000 bp-long homologous arms to the *PRNP* gene. Upon targeted integration, the PrP ORF is replaced by the GFP ORF, where GFP expression will be driven by the promoter of the *PRNP* gene. The As- and LbCpf1 plasmids containing the four crRNAs targeting *PRNP* used in the previous experiment (Fig. 3a) were cotransfected with the donor plasmid and the number of GFP expressing cells were monitored. Interestingly, transfection of the promoterless donor plasmid with the inactive dSpCas9 expressing plasmid resulted in some GFP fluorescence when measured two days after transfection. We used this transient fluorescent signal hereafter in the experiments to compare the transfection efficiencies of different samples using identical donor plasmid. These transient signals decayed in about six to nine days, after that allowing the measurement of the GFP signal originating from the integrated GFP cassettes.

**Fig. 5:**
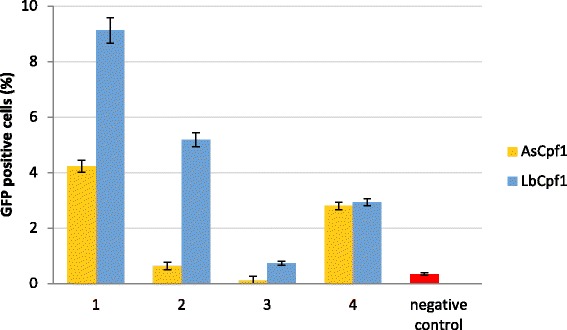
As- and LbCpf1 induced HDR at different genomic cleavage sites. Percentages of GFP fluorescent cells after HDR mediated integration of a promoterless donor GFP cassette. The efficiencies of both As- and LbCpf1 nucleases (orange and blue, respectively) to induce HDR mediated integration were tested on four mouse PrP genomic targets (PRNP 1-4, same targets as present on Fig. 3a). The nuclease vector and the homologous recombination donor molecule were cotransfected into N2a cells. As negative control, cells were transfected with the donor molecule and an inactive SpCas9 expression plasmid. On the seventh day after transfection GFP positive cells were counted. Three parallel transfections were made for each sample. Two days after transfection all of the samples showed similar GFP positive cell counts. Both Cpf1 nucleases mediated homology directed integration of the donor cassette in N2a cells

The result on Fig. 5 show that both nucleases mediate homology directed integration of the donor cassette in N2a cells. Among the four *PRNP* targets tested, LbCpf1 cleaved three, whereas AsCpf1 cleaved two targets that resulted in fluorescence by an order of magnitude higher than the level of the negative control that corresponds to the random integration of the donor molecule. We also found similarly low levels of fluorescence with controls where the Cpf1 nucleases were cotransfected with a plasmid containing no corresponding homology arms (data not shown), indicating that Cpf1’s activity does not result any significant fluorescence originating from non-HDR mediated on-target and random integration. This is likely due to the fact that no promoter precedes the GFP cassette, thus its integration is silent unless it becomes integrated downstream of a promoter. The integration of the donor DNA also depends on other factors than the nuclease cleavage efficiencies. One important factor is the position of the target relative to the homologous arms. This may explain the somehow lower integration efficiency at site PRNP 4 (number 4 on Fig. 5) of which position is the most remote from the homologous arms. Interestingly, LbCpf1 seems to be slightly more active in this assay mediating integrations two to four fold higher at three out of the four targets than AsCpf1 (Fig. 5). We found similar integration efficiencies of the same donor plasmid when using Sa- and StCas9 on a few targets (Additional file 6); however, a strict comparisons to the Cpf1 nucleases is precluded due to the different distances of their targets from the homologous arms that is the consequence of the rare occurrences of their 4-5 nucleotide-long PAM sequences. Our attempts to mediate integration of the donor plasmid using NmCas9 with a few targets repeatedly failed.

## Discussion

The results presented here significantly extend our knowledge on Cpf1 nucleases: these are the first demonstration that Cpf1 nucleases induce efficient DNA recombination in mammalian cells based on homologous sequences and exploring N2a cells – a different cell line – as compared to human HEK293FT cells used in the pioneering studies of Zetsche et al. Double strand DNA breaks are generally repaired by one of the main repair pathways, NHEJ or HR. The factors affecting the choice between the two pathways are not fully understood; however, they include both the types of the DNA break and of the host cells [38]. SpCas9 nuclease has been reported to remain bound to the DNA target sites after cleavage for an extended period of time [39] that is likely to significantly affect the choice of repair pathways. It is not known if Cpf1 nucleases also remain bound to their DNA substrates after cleavage or not. Based on the available X-ray structure [25, 27, 40] and on their cleavage position, it is likely that a Cpf1 nuclease would not keep the cleaved DNA ends together after cleavage, even if it remains bound to the DNA. How this distinct feature of Cpf1 nucleases would affect the repair pathway choice, it is not known. Nevertheless, our data show that Cpf1 nucleases are able to induce efficient HR repair in mammalian cells.

During DNA break repair, SSA and HR repair pathways are initiated by identical mechanism and are being separated only after the step of extensive 3’ end resections of the DNA that also blocks the NHEJ repair pathways [38, 41]. Although the HR repair pathway, exploiting homology arms on a donor plasmid is employed more often, here, the SSA pathways are exploited to interrogate the activities of Cpf1 nucleases for the following reasons. HR repair is heavily influenced by the distances of the targets from the homology arm. To avoid this effect when a number of nucleases are compared, targets with the corresponding PAM sequences need to be found at nearly identical genomic positions. However, this is far from being a trivial task because several of these nucleases require at least three-nucleotide-long PAM sequences (As-, LbCpf1, Nm-, Sa-, StCas9). In addition, carrying out a HR assay takes about nine to fourteen days while conducting a GFxFP assay requires only four days that presents advantages.

Our results not only show that the two Cpf1 nucleases are as or more effective than the most widely used Cas9 counterparts of SpCas9, but also confirm the superior utility of SpCas9 and the somewhat lower activity of NmCas9 to that of Sa- and StCas9s. These results are consistent with our previous characterization of these Cas9 nucleases (data not shown).

Even more significant is the demonstration of the robust activity of Cpf1 nucleases in mammalian cells. In the experiments here (Fig. 3 and Additional file 2: Table S3), 19 sequences out of the targeted 20 were cleaved efficiently by both Cpf1 nucleases inducing SSA repair. The activities of Cas9 nucleases that are characterized so far vary more widely among various target sequences. A thorough characterization of Cpf1 nucleases’ target specificity, like in the case of SpCas9, may further lead to improve their effectiveness.

## Conclusions

While examining only a limited number of target sequences, our results suggest that As- and LbCpf1 nucleases, although being less utile than SpCas9 in these experiments, are able to induce homology directed repair (SSA and HR) in N2a cells. They demonstrate especially robust activity to induce SSA in the plasmid-based GFxFP assay and are equally or more effective than the most frequently used orthogonal Cas9 counterparts of SpCas9. These results suggest an important position for the Cpf1 nucleases on the palette of effective genome engineering tools on the side of SpCas9.

## Methods

### Materials

Restriction enzymes, Klenow polymerase, T4 ligase and Pfu polymerase were purchased from Thermo Fischer Scientific. DNA oligonucleotides were acquired from Microsynth AG [42] or Sigma-Aldrich Co. All DNA constructs made were verified by sequencing by Microsynth AG. Plasmids were purified with GenElute HP Plasmid Miniprep kit (Sigma-Aldrich). Q5 polymerase was from New England BioLabs Inc. Dulbecco’s modified Eagle’s Medium, foetal bovine serum, Turbofect, penicillin and streptomycin were acquired from Thermo Fisher Scientific. The following plasmids were acquired from Addgene: piRFP670-N1 #45457 [32]; pX330-U6-Chimeric_BB-CBh-hSpCas9 #42230 [4]; pCAG-EGxxFP #50716 [31]; pY010 (pcDNA3.1-hAsCpf1) #69982 [24]; pY016 (pcDNA3.1-hLbCpf1) #69988 [24]; M-ST1cas #48669 [23]; and pSimpleII-U6-tracr-U6-BsmBI-NLS-NmCas9-HA-NLS(s) #47868, [35].

### Plasmid construction

Vectors were constructed using standard molecular biology techniques. For detailed information see Additional file 7. The sequences of DNA oligonucleotides used in these studies are listed in Additional file 2.

### Cell culturing and transfection

N2a cells (Neuro-2a mouse neuroblastoma cells, ATCC – CCL-131) were grown at 37 °C in a humidified atmosphere of 5 % CO_2_ in high glucose Dulbecco’s Modified Eagle medium (DMEM) supplemented with 10 % heat inactivated fetal bovine serum, 4 mM L-glutamine (Gibco), 100 units/ml penicillin and 100 μg/ml streptomycin.

#### Details of GFxFP assay

Cells cultured on 48-well plates, were seeded a day before transfection at a density of 3 × 10^4^ cells/well. The next day, at around 40 % confluence, cells were transfected with plasmid constructs using Turbofect reagent, briefly as follows: 250 ng total plasmid DNA and 1 μl Turbofect were mixed in 50 μl serum free DMEM and the mixture was incubated for 30 min at room temperature prior adding to cells. Transfection medium was changed on the cells to fresh supplemented DMEM after 24 h of incubation. Three parallel transfections were made from each sample. A mCherry expression plasmid was cotransfected with each sample to monitor the transfection efficiency. Cells were analyzed by flow cytometry two days after transfection.

#### Details of genomic HDR assay

Cells cultured on 6-well plates were seeded a day before transfection at a density of 10^5^ cells/well. The next day, at around 30 % confluence, cells were transfected with plasmid constructs using Turbofect reagent as follows: 4000 ng total plasmid DNA and 4 μl Turbofect was mixed in 400 μl serum free DMEM and the mixture was incubated for 30 min at room temperature prior adding to cells. Transfection medium on the cells was changed to fresh supplemented DMEM after 24 h of incubation. Three parallel transfections were made for each sample. Two days after transfection, cells were trypsinized and divided as follows: 10 % of the cells were seeded on new 6-well plates and 90 % of the cells were analyzed by flow cytometry. Seven days after transfection samples were measured again by flow cytometry.

### Flow cytometry

Flow cytometry analysis was carried out on Attune Acoustic Focusing Cytometer (Applied Biosystems by Life Technologies) and on BD FACSCanto II [Becton Dickinson Immunocytometry Systems]. For data analysis Attune Cytometric Software was used. In all experiments, a total of 10,000 viable single cells were acquired and were gated based on side and forward light-scatter parameters. Cells expressing GFP and mCherry from a control plasmid were used to identify GFP and mCherry positive cells in the samples. The GFP signal was detected using the 488 nm diode laser for excitation and the 530/30 nm filter for emission. The mCherry signal was detected using the 488 nm diode laser for excitation and a 640LP filter for emission. The signal of iRFP670 (near-infrared fluorescent protein with emission maxima at 670 nm) was detected using the 633 nm diode laser for excitation and the 660/20 nm filter for emission.

## Reviewers’ comments

**Author’s response*:****We thank all three reviewers for accepting reviewing our manuscript and for their comments/recommendations that helped to improve the manuscript. We also thank all three reviewers for the thorough reviewing of the text; for spotting typos, omissions and inaccuracies, that helped us in editing and improving the manuscript.*

### Reviewer’s report 1

Eugene Koonin, NCBI, NLM, NIH, USA

## Reviewer comments

In the article titled “Cpf1 nucleases demonstrate robust activity to induce DNA modification by exploiting homology directed repair pathways in mammalian cells”, Toth et al describe target cleavage and induction of homology-directed repair in human cells by two RNA-guided nucleases of the Cpf1 family. The results suggest that both Cpf1 proteins are capable of efficient target cleavage, being apparently less active than the most commonly used SpCas9 but about as active as other characterized Cas9 species. The work extends the existing knowledge on Cpf1, potentially an important, novel genome editing tool.

The work is somewhat preliminary in the sense that only two Cpf1 species have been studied, off-target effects were not assessed, and the comparison to SpCas9 cannot be considered reliable because for SpCas9, the guide RNA were designed whereas for the rest of the studied proteins, the guides were chosen randomly. It is hard to tell why the experiments were done in this non-uniform manner. It should not be difficult to use random guides with SpCas9. Without that simple experiment, the conclusion that Cpf1 is less effective than SpCas9 does not seem to be quite justified.

**Author’s response**: *The reviewer is right that the work would be more comprehensive by studying more Cpf1 nucleases. However, Zetsche* et al. *showed that only these two Cpf1 species have considerable activity in mammalian (HEK293FT) cells. We agree that it is likely interesting to extend these studies on other Cpf1 species and by using more kinds of mammalian systems and we plan to do so.*

*He is also right in his comment on the differential ways we selected our targets using prediction tools only for SpCas9, which makes less fair the comparison of their natural activities. However, our attempt was different here. Although the sequence specificity of SpCas9 is not yet fully understood and the prediction of spacer efficiency is yet at an immature stage at present, SpCas9 is the only RNA-guided nuclease for which such prediction tools are available. Contrary, Cpf1 nucleases and the other Cas9s are much less studied and so far we know very little about their specificities as compared to SpCas9. Thus, from a practical point of view when one needs to decide which nucleases to choose to efficiently carry out genome modification experiments, currently, SpCas9 being supported with better spacer prediction may stand against the use of a Cpf1 nuclease where spacer sequence efficiency selection is not supported yet by spacer efficiency prediction tools, leaving the only option of random selection from possible targets. It is this situation where our experiments might provide valuable guidance. Nevertheless, when more knowledge is gained on Cpf1 nucleases based on which spacer efficiency prediction tools can be built, this issue may need to be revisited. We discuss and emphasize more this issue, highlighted by the reviewer, in our revised manuscript.*

### Reviewer’s report 2

Haruhiko Siomi, Keio University School of Medicine, Japan

## Reviewer comments

Recently Cpf1 has been established as a class 2 CRISPR-Cas system that includes an effective single RNA-guided endonuclease (Zetsche et al. Cell 2015). This study compares the single strand annealing (SSA) activity of two members of the Cpf1 family, the AsCpf1 and the LbCpf1 with that of various Cas9 proteins. The authors find that both AsCpf1 and LbCpf1 appear to mediate SSA repair activity more efficiently than three of Cas9 counterparts do; SaCas9, StCas9 and NmCas9. However, SpCas9 mediates SSA repair more efficiently than the two Cpf1 proteins. These Cpf1 proteins are potentially powerful tools to edit genomes, but the paper falls short of clearly demonstrating the usefulness of the SSA activity mediated by Cpf1 proteins.

Major points:

1. The authors should, at least, describe potential useful features of the SSA mediated by Cpf1 proteins for genome editing.”

**Author’s response***: SSA mediated repair can be useful when direct repeats are to be edited exploiting designer nucleases, among them Cpf1s. However, our point using SSA repair here is different. We aim to compare the ability of Cpf1 and Cas9 nucleases to induce homology directed repair (HDR),* i.e. *repair mechanisms based on homologous sequences involving either homologous recombination (HR) or single strand annealing (SSA).*

*During DNA break repair, SSA and HR repair pathways are initiated by identical mechanism and are being separated only after the step of extensive 3’ end resections of the DNA ends that also block the NHEJ repair pathway. Thus, the differing ability of Cas9 and Cpf1 nucleases (emerging from differences in the nature of the double strand breaks and in their interaction with the target DNA) to allow the engagement of the repair system for 3’ end resections may be well revealed by monitoring either the SSA or the HR repair pathways.*

*Although the HR repair pathway, exploiting homology arms on a donor plasmid is employed more often, here, the SSA pathway is also exploited to interrogate the activities of Cpf1 nucleases to induce HDR for the following reason: HR repair is heavily influenced by the distances of the targets from the homology arm. To avoid the contribution of this effect when two or more nucleases (with different PAM sequences) are compared, the targets need to be found at nearly identical genomic positions in relation to the homology arms. For this, the corresponding PAM sequences need to be found at nearly identical positions. However, this is far from being trivial because several of these nucleases require at least three to five nucleotide-long PAM sequences (As- and LbCpf1 three, Nm-, Sa-, StCas9 four to five), which are rather infrequent.*

*Nevertheless, we also monitored here how Cpf1 nucleases induce HR repair, although a very strict comparison cannot be carried out with Cas9s from the above discussed reasons.*

2. The authors shall consider performing experiments with cleavage-dead Cpf1 in Figs. 3, 4, and 5.

**Author’s response***: In respect to Figs.*3*and*4*an active Cpf1 nuclease without crRNA, whereas on Fig.*5*a dead SpCas9 was used as negative control. We did generate the corresponding dead As- and LbCpf1 nucleases; however, at the time the experiments were carried out we had not characterized them whether they are really fully inactive or not. Now, we detect no difference between these three types of controls, dead Cpf1, SpCas9 and active As- and LbCpf1s without crRNAs, therefore, they can be used in an interchangeable manner.*

3. It seems the authors take the increase in the number of GFP positive cells as read-out of the increase in the efficiency of cleavage. But the expression of GFP in their assay involves cleavage and homologous recombination. These are in essence totally different steps.

**Author’s response**: *We attempted to discern the ability of these nucleases to “induce homology directed repair”. There is no experimental tool currently available to assess directly the in-cell cleavage activity of a nuclease. The closest equivalent may be the BLESS (direct in situ breaks labeling, enrichment on streptavidin and next-generation sequencing) method (N Crosetto* et al.*, Nature Methods, 2013) that captures the double strand breaks that are present at a given moment in the cells. The “efficiency” of a nuclease is generally inferred based on detecting the outcome, generally the alteration in the DNA sequence as a direct result of the cell DNA repair mechanism that is measured by various readouts. As such, the cleavage activities may be different than assessed by relaying on the outcome of different repair mechanisms. Plausibly, this is the point the reviewer refers to - and it is the point of the manuscript as well. We try to assess here the ability of these nucleases to induce homology directed repair, SSA and HR, contrary of to induce NHEJ-mediated repair. To avoid confusions, as pointed by the reviewer, we have changed the wording of corresponding lines in the manuscript to clarify these points.*

### Reviewer’s report 3

Jean-Yves Masson, Universitè Laval, Canada

## Reviewer comments

In this manuscript, E. Toth and colleagues aim to characterize the efficiency of two Cpf1 family members (AsCpf1 and LbCpf1) in genome engineering. These nucleases have been characterized previously for indel frequencies by non-homologous end joining (Zetsche et al., Cell 163, 759-771). However, their activities on homology directed repair and single-strand annealing remained unclear. This is an important question in a fast growing field where the most efficient tools must be used for genome engineering. They also used another cell line, N2a cells, which is different from the studies of Zetsche et al.

Overall, this nice study suggests that AsCpf1 and LbCpf1 are alternative tools to Cas9. In fact, they are more efficient to induce SSA than Cas9 homologs. Moreover, these nucleases are very efficient, showing cleavage of 19 out of 20 loci.

Figure 1 and Additional file 1: Figure S1B. It is surprising that ~ 30 % of the control cells display GFP fluorescence without any nuclease cleavage. What is the level of endogenous DSBs in the N2a cells? This can be monitored with immunofluorescence staining with g-H2AX and 53BP1.

**Author’s response**: *This value is actually 10 to 20 % when the GF-chl-FP constructs are used (Fig.*1*b) where recombination events that possibly occur in Escherichia coli during plasmid-generation is minimized. Such background fluorescence obtained in N2a cells can also be observed at a similar extent in HEK293T and at a lower extent in HeLa cells (Additional file*3*). The higher fluorescence apparent also after nuclease cleavage may indicate that the complex process starting from the SSA repair of the DNA break and extending to the detection of the fluorescent signal of the expressed GFP protein, may somehow more efficiently take place in N2a cells. However, a contribution from a somewhat higher level of endogenous DSBs neither can be ruled out, as also evidenced in Additional file*3*.*

What is the percentage of cells that are transfected with mCherry? It is not clear whether SSA is low because of the transfection efficiency. The transfection efficiency for each experiment should be shown.

**Author’s response**: *The results that are shown on Fig.*2*c are normalized to the corresponding mCherry positive population in case of each sample. In the revised version of the manuscript we added this information, previously present in the materials and methods section, to the main text too in order to clarify this issue. We did a few attempts to increase the efficiency of the Cpf1 nucleases using the PCR products, however, without much success. The transfection efficiencies were relatively similar in these experiments: 55.9 % +/- 10.5 % when the plasmid was used, whereas 45.0 % +/- 8.4 %, when the PCR product. Now we indicate these values in the corresponding figure legend for clarity.*

The authors should make a clear distinction between SSA and HDR. SSA is a RAD52-dependent pathway and HDR relies mostly on BRCA1-PALB2-BRCA2 and RAD51. For instance, SSA is monitored in Fig. 4, but the authors mention in the text that « Cpf1 nucleases were equally or more effective in inducing HDR on average than the three SpCas9 counterparts ». This is confusing with Fig. 5. In Fig. 5, a typical HDR assay is employed (with an homologous recombination donor). The authors should carefully edit the manuscript to distinguish SSA assays from HDR assays.

**Author’s response**: *There is some inconsistency in the literature regarding the terminologies used. We refer as homology directed repair (HDR) to the repair pathways that are based on homologous sequences, involving both homologous recombination (HR) and single strand annealing (SSA), as it is frequently used in the literature (A Ciccia and SJ Elledge, Mol Cell, 2010; G Soria and G Almouzni, Cell Cycle, 2013; F Larminat* et al.*, Oncogene, 2002; M Keimling and L Wiesmüller, Carcinogenesis, 2009). We also noticed papers that use HR and HDR in the opposite sense [HR involving both HDR and SSA pathways (SM Howard, DA Yanez and JM Stark, PLoS Genet, 2015)] or which use other terms, such as conservative or inter molecular HDR and homology repair [also denoted as HR, (L Cong* et al.*, Science, 2013)] for the pathway relying mostly on BRCA1-PALB2-BRCA2 and RAD51 [as opposed to homologous recombination between direct repeats (RAD52-dependent]). This is clearly indicated now at the end of the background section.*

Also, in this particular experiment, SaCas9, StCas9 and NmCas9 should be used as controls. This would help the reviewer to conclude whether they are indeed better tools for HDR.

**Author’s response**: *We had performed HR experiments with Sa- and StCas9s (results now presented in Additional File*6*); however, the target positions of the Cas9s generally do not match those of the Cpf1s, which in our judgment precludes their use for a fair comparison (relative distance from the cleavage position to the homology arms might affect the efficiency of homologous repair-based integration). This was one of the reasons we preferred to use the GFxFP plasmid system where the infrequent presence of the different PAM sequences doesn’t impede the comparison of the efficiencies of different nucleases.*

To make a more comprehensive study, the authors should use also integrated reporter cassettes.

**Author’s response**: *We also considered the approach of using an integrated GFP reporter cassette. Unfortunately, in contrast to SpCas9 having a two nucleotide-long PAM restriction (GG), Lb- and As-Cpf1 and Sa-, St-and NmCas9 nucleases have longer, 3 to 5 nucleotide-long PAM sequences and thus, much less targetable sequences are available. As a result, Lb- and AsCpf1 (PAM: TTT) nucleases have only two targets, while StCas9 (PAM: AGAAW) and NmCas9 (PAM: GATT) have no target in the GFP reporter construct. Considering mCherry, it has only one position that has more than two consecutive T nucleotides. The construction of a new cell line harboring an integrated reporter cassette of which sequences are altered in order to contain a reasonable number of PAM sequences in question was out of the time frame of this study.*

They should also make an attempt to discuss off-target events in light of the two recent studies below:

Daesik Kim, Jungeun Kim, Junho K Hur, Kyung Wook Been, Sun-heui Yoon & Jin-Soo Kim. Genome-wide analysis reveals specificities of Cpf1. Nature Biotechnology, June 2016 doi:10.1038/nbt.3609.

Junho K Hur, Kyoungmi Kim, Kyung Wook Been, Gayoung Baek, Sunghyeok Ye, Junseok W Hur, Seuk-Min Ryu, Youn Su Lee & Jin-Soo Kim. Targeted mutagenesis in mice by electroporation of Cpf1 ribonucleoproteins. Nature Biotechnology, June 2016 doi:10.1038/nbt.3596.

**Author’s response**: *We included a short discussion about the fidelity of these Cpf1 nucleases based on the more recent publications in the Background section.*

I acknowledge the spirit of the authors, for deposition of AsCpf1 and LbCpf1 plasmids at Addgene.

## Additional files

Additional file 1: Figure S1. GFxFP reporter assay. (DOCX 766 kb)

Additional file 2: **Table S1, Table S2 and Table S3.** Oligonucleotide used in the study. (DOCX 35 kb)

Additional file 3: Figure S2. The origin of background fluorescence of GFxFP plasmids. (DOCX 2420 kb)

Additional file 4: Figure S3. Schematic structure of As- (pTE4396) and LbCpf1 (pTE4398) expression plasmids with crRNA expression cassettes. (DOCX 99 kb)

Additional file 5: Figure S4. LbCpf1 exert higher efficiency to induce HDR than three tested Cas9 counterparts. (DOCX 143 kb)

Additional file 6: Figure S5. Homologous recombination of a GFP casette mediated by St- or SaCas9 at the PRNP locus. (DOCX 13 kb)

Additional file 7: Supplementary Materials and Methods. (DOCX 41 kb)

## Abbreviations

As: *Acidaminococcus sp. BV3L6*
CAG: CMV early enhancer/chicken β actin promoter
Cas: CRISPR-associated protein
Chl: Chloramphenicol resistance gene expression cassette
Cpf1: CRISPR from *Prevotella* and *Francisella* 1
CRISPR: Clustered regularly interspaced short palindromic repeat
crRNA: CRISPR RNA
dCas9: Dead or nuclease inactive *Streptococcus pyogenes* Cas9
DMEM: Dulbecco’s modified Eagle Medium
DNMT: DNA methyltransferase
Fn: *Francisella novicida U112*
GFP: Green fluorescent protein
gRNA: Guide RNA
HDR: Homology directed repair
HR: Homologous recombination
iRFP670: Near-infrared fluorescent protein with emission maxima at 670 nm
Lb: *Lachnospiraceae bacterium ND2006*
MCS: Multiple cloning site
mDpl: Mouse doppel protein
mPrP: Mouse prion protein
mSho: Mouse shadoo protein
N2a: Neuro-2a mouse neuroblastoma cell line
NHEJ: Non-homologous end joining
Nm: *Neisseria meningitidis*
ORF: Open reading frame
PAM: Protospacer adjacent motif
*PRND*: Gene of doppel protein
*PRNP*: Gene of prion protein
PrP: Prion protein
Sa: *Staphylococcus aureus*
Sp: *Streptococcus pyogenes*
*SPRN*: Gene of shadoo protein
SSA: Single strand annealing
St: *Streptococcus thermophilus*
SV40: Simian vacuolating virus 40
tracrRNA: Transactivating CRISPR RNA
