# Supplementary material for: Cpf1 nucleases demonstrate robust activity to induce DNA modification by exploiting homology directed repair pathways in mammalian cells
Source: Biol Direct. 2016 Sep 14;11:46. doi: 10.1186/s13062-016-0147-0 (PMC5024423; doi:10.1186/s13062-016-0147-0)
Supplement: Supplementary file 1 — GFxFP reporter assay. (DOCX 766 kb) [file 13062_2016_147_MOESM1_ESM.docx]

**Additional file 1**

**Figure S1. GFxFP reporter assay**

**A.)** Schematics of the principle of the reporter assay used for monitoring the activity of nucleases. The sequences of the two GFP halves are indicated in green. Each of them contains both a non-overlapping segment (dark green), and an overlapping segment (light green). The expression cassette is driven by a CAG promoter (grey arrow) and terminated by a SV40 (Simian Vacuolating Virus 40) polyA signal (pA, grey box). Expression from the first GFP half is terminated by a STOP codon (black mark), and thus, results in no detectable green fluorescence. The second GFP half has neither Kozak sequence nor ATG. The nuclease target site (blue box) is to be cloned to a multicloning site (MCS, yellow) between the two GFP halves. Upon nuclease cleavage at the target site the generated double-strand DNA break is repaired by single strand annealing directed by the overlapping homologous sequences. As a result an intact GFP sequence is created from which an active GFP is transcribed. **B.)** The percentages of cells exhibiting GFP fluorescence are shown. PrP10 and Sp1 denote the cell populations where the plasmid with the interrupted GFP sequences containing either the PrP10 or the Sp1 target was cotransfected with the corresponding gRNA- and the active nuclease-expressing vector. dSpCas9 refers to the control cells cotransfected with an inactive SpCas9-expressing vector. Values are normalized to the transfection efficiency measured by exploiting the fluorescence of iRFP670. Error bars show the mean ± standard deviation of percentages measured in N=3 independent transfections. **C.)** Representative histograms of GFP fluorescence intensities measured by flow cytometry analysis of PrP10, Sp1 and dSpCas9 samples at day two posttransfection and of an untransfected cell population.
