# Supplementary material for: Cpf1 nucleases demonstrate robust activity to induce DNA modification by exploiting homology directed repair pathways in mammalian cells
Source: Biol Direct. 2016 Sep 14;11:46. doi: 10.1186/s13062-016-0147-0 (PMC5024423; doi:10.1186/s13062-016-0147-0)
Supplement: Supplementary file 2 — Table S1, Table S2 and Table S3. Oligonucleotide used in the study. (DOCX 35 kb) [file 13062_2016_147_MOESM2_ESM.docx]

**Additional file 2**

**Supplementary Tables**

**Table S1. Oligonucleotides used for SpCas9, SaCas9, NmCas9 and StCas9 spacer cloning.** Oligonucleotide sequences and acceptor vectors used for spacer cloning are listed.

| **Spacer** | **Cas9 acceptor vector** | **Oligonucleotide used*** |
| --- | --- | --- |
| **Sp1** | SpCas9_IRFP | *CACC*GTCTCCGAAGGAACGTGTCA |
|  |  | *AAAC*TGACACGTTCCTTCGGAGAC |
| **PrP10** | SpCas9_IRFP | *CACC*GTCCTGATCGTGGGATGAGGG |
|  |  | *AAAC*CCCTCATCCCACGATCAGGAC |
| **Sp2** | px330-U6-Chimeric_BB-CBh-hSpCas9 (1) | *CACC*GTCAGTCATCATGGCGAACCT |
|  |  | *AAAC*AGGTTCGCCATGATGACTGAC |
| **Sp3** | px330-U6-Chimeric_BB-CBh-hSpCas9 (1) | *CACC*GATTTTGCAGATCAGTCATCA |
|  |  | *AAAC*TGATGACTGATCTGCAAAATC |
| **Sp4** | px330-U6-Chimeric_BB-CBh-hSpCas9 (1) | *CACC*GTCCTGATCGTGGGATGAGGG |
|  |  | *AAAC*CCCTCATCCCACGATCAGGAC |
| **Sp5** | px330-U6-Chimeric_BB-CBh-hSpCas9 (1) | *CACC*GCCATGATGACTGATCTGCAA |
|  |  | *AAAC*TTGCAGATCAGTCATCATGGC |
| **Sp6** | px330-U6-Chimeric_BB-CBh-hSpCas9 (1) | *CACC*GAATTCACCGAGCAGGAGTGA |
|  |  | *AAAC*TCACTCCTGCTCGGTGAATTC |
| **Sa1** | px601-AAV-CMV::NLS-SaCas9-NLS-3xHAbGHpA;U6::Bsa-SgRNA (2) | *CACC*GCCATGATGACTGATCTGCAAA |
|  |  | *AAAC*TTTGCAGATCAGTCATCATGGC |
| **Sa2** | px601-AAV-CMV::NLS-SaCas9-NLS-3xHAbGHpA;U6::Bsa-SgRNA (2) | *CACC*GCAAAAAGCGGCCAAAGCCTG |
|  |  | *AAAC*CAGGCTTTGGCCGCTTTTTGC |
| **Sa3** | px601-AAV-CMV::NLS-SaCas9-NLS-3xHAbGHpA;U6::Bsa-SgRNA (2) | *CACC*GTCATCTTCCTGATCGTGGGAT |
|  |  | *AAAC*ATCCCACGATCAGGAAGATGAC |
| **Sa4** | px601-AAV-CMV::NLS-SaCas9-NLS-3xHAbGHpA;U6::Bsa-SgRNA (2) | *CACC*GCATCTTCCTGATCGTGGGATG |
|  |  | *AAAC*CATCCCACGATCAGGAAGATGC |
| **Sa5** | px601-AAV-CMV::NLS-SaCas9-NLS-3xHAbGHpA;U6::Bsa-SgRNA (2) | *CACC*GATCTTCCTGATCGTGGGATGA |
|  |  | *AAAC*TCATCCCACGATCAGGAAGATC |
| **Sa6** | px601-AAV-CMV::NLS-SaCas9-NLS-3xHAbGHpA;U6::Bsa-SgRNA (2) | *CACC*GTTTTGCAGATCAGTCATCATG |
|  |  | *AAAC*CATGATGACTGATCTGCAAAAC |
| **St1** | pTE4254-StCas9 | *AAAC*AGATCAGTCATCATGGCGAAc |
|  |  | *CACC*GTTCGCCATGATGACTGATCT |
| **St2** | pTE4254-StCas9 | *AAAC*GTGACTATGTGGACTGATGTC |
|  |  | *CACC*GACATCAGTCCACATAGTCAC |
| **St3** | pTE4254-StCas9 | *AAAC*TCCCCTCCTGTCATCCTCCTC |
|  |  | *CACC*GAGGAGGATGACAGGAGGGGA |
| **St4** | pTE4254-StCas9 | *AAAC*TCGTGGGATGAGGGAGGCCTC |
|  |  | *CACC*GAGGCCTCCCTCATCCCACGA |
| **St5** | pTE4254-StCas9 | *AAAC*ATCCCACGATCAGGAAGATGC |
|  |  | *CACC*GCATCTTCCTGATCGTGGGAT |
| **St6** | pTE4254-StCas9 | *AAAC*TCCCCTCCTGTCATCCTCCTC |
|  |  | *CACC*GAGGAGGATGACAGGAGGGGA |
| **Nm1** | pVB7107-NmCas9 | *CAAC*TGATGACTGATCTGCAAAATGAGGC |
|  |  | *CACC*GCCTCATTTTGCAGATCAGTCATCA |
| **Nm2** | pVB7107-NmCas9 | *CAAC*ATCATGGCGAACCTTGGCTACTGGC |
|  |  | *CACC*GCCAGTAGCCAAGGTTCGCCATGAT |
| **Nm3** | pVB7107-NmCas9 | *CAAC*TCGTGGGATGAGGGAGGCCTTCCTC |
|  |  | *CACC*GAGGAAGGCCTCCCTCATCCCACGA |
| **Nm4** | pVB7107-NmCas9 | *CAAC*GATGAGGGAGGCCTTCCTGCTTGTC |
|  |  | *CACC*GACAAGCAGGAAGGCCTCCCTCATC |
| **Nm5** | pVB7107-NmCas9 | *CAAC*AGGCCTCCCTCATCCCACGATCAGC |
|  |  | *CACC*GCTGATCGTGGGATGAGGGAGGCCT |

*: The overhangs of the DNA linkers are marked in italics.

**Table S2. Oligonucleotides used for nuclease target cloning into GFxFP vectors.**

The names of the targets, the acceptor vectors and the sequences of the corresponding oligonucleotides used are listed. Two targets are generally incorporated into one oligonucleotide linker.

| **Target** | **GFxFP vector** | **Oligonucleotide used*** |
| --- | --- | --- |
| **Sp1, Prp10** | pEGxxFP (3), pGF-chl-FP, pGF-ori-FP | *GATC*CTTCCTCATCTTCCTGATCGTGGGATGAGGGAGGCCTTCCTGCTTTCCAGTCTCCGAAGGAACGTGTCACGGTTGTAG |
|  |  | *AATT*CTACAACCGTGACACGTTCCTTCGGAGACTGGAAAGCAGGAAGGCCTCCCTCATCCCACGATCAGGAAGATGAGGAAG |
| **DNMT1.3** | pGF-ori-FP | *GATC*CACTCCCGTCTTCGATATCGAAGACCCTTTCCTGATGG TCCATGTCTGTTACTCGGAGACGAAGCTTCGTCTCGTTTCG |
|  |  | *AATT*CGAAACGAGACGAAGCTTCGTCTCCGAGTAACAGACAT GGACCATCAGGAAAGGGTCTTCGATATCGAAGACGGGAGTG |
| **1, 12** | pGF-ori-FP | *GATC*GCCTTTGCAGATCAGTCATCATGGCGAACCTTGGAAAA ATTGGAGACGAAGCTTCGTCTCCGATCC |
|  |  | *AATT*GGATCGGAGACGAAGCTTCGTCTCCAATTTTTCCAAGGT TCGCCATGATGACTGATCTGCAAAGGC |
| **2, 13** | pGF-ori-FP | *GATC*GCCCTCTTTGTGACTATGTGGACTGATGTCGGCAAATTG GAGACGGTCGACCGTCTCCGATC |
|  |  | *AATT*GATCGGAGACGGTCGACCGTCTCCAATTTGCCGACATC AGTCCACATAGTCACAAAGAGGGC |
| **3, 14** | pGF-ori-FP | *GATC*TTTATGTCGGCCTCTGCAAAAAGCGGCCAAAGCAATTG GAGACGCTGCAGCGTCTCCGATC |
|  |  | *AATT*GATCGGAGACGCTGCAGCGTCTCCAATTGCTTTGGCCGC TTTTTGCAGAGGCCGACATAAA |
| **4, 15** | pGF-ori-FP | *GATC*TTTCCAAGGAGGGGGTACCCATAATCAGTGGAACAAGC CCAGCAAAAATTGGAGACGACGCGTCGTCTCCGATC |
|  |  | *AATT*GATCGGAGACGACGCGTCGTCTCCAATTTTTGCTGGGCT TGTTCCACTGATTATGGGTACCCCCTCCTTGGAAA |
| **5, 16** | pGF-ori-FP | *GATC*GCCACGTATTTGTCTGCTTCTGGGCGGCACCCTTGGAAA TTGGAGACGGATATCCGTCTCCGATC |
|  |  | *AATT*GATCGGAGACGGATATCCGTCTCCAATTTCCAAGGGTGC CGCCCAGAAGCAGACAAATACGTGGC |
| **6, 17** | pGF-ori-FP | *GATC*TTTCCCTTGCAGATTCACCATGAAGAACCGGCTGGAAAT TGGAGACGTTCGAATCGTCTCCGATC |
|  |  | *AATT*GATCGGAGACGATTCGAACGTCTCCAATTTCCAGCCGGT TCTTCATGGTGAATCTGCAAGGGAAA |
| **7, 18** | pGF-ori-FP | *GATC*TTTCTCTCCACGGTCAAGGCAAGGGGCATAAATTGGAGA CGCCCGGGCGTCTCCGATC |
|  |  | *AATT*GATCGGAGACGCCCGGGCGTCTCCAATTTATGCCCCTTG CCTTGACCGTGGAGAGAAA |
| **8, 19** | pGF-ori-FP | *GATC*TTTCCGCCAAGCACTGCGATTTCTGGCTGGAAATTGGAG ACGGAGCTCCGTCTCCGATC |
|  |  | *AATT*GATCGGAGACGGAGCTCCGTCTCCAATTTCCAGCCAGAA ATCGCAGTGCTTGGCGGAAA |
| **9, 20** | pGF-ori-FP | *GATC*GCCTTTCTGGCTGGAAAGGGGAGCTGCGCTTCGGAAATT GGAGACGAGGCCTCGTCTCCGATC |
|  |  | *AATT*GATCGGAGACGAGGCCTCGTCTCCAATTTCCGAAGCGCA GCTCCCCTTTCCAGCCAGAAAGGC |
| **10, 11** | pGF-ori-FP | *GATC*GCCGTTTCGTTTGGTTCATTGTGAAGTAAAGGAATTGGA GACGGCTAGCTCGTCTCCGATC |
|  |  | *AATT*GATCGGAGACGAGCTAGCCGTCTCCAATTCCTTTACTTC ACAATGAACCAAACGAAACGGC |
| **mPrP ATG** | pGF-ori-FP | *GATC*CCCTCATTTTGCAGATCAGTCATCATGGCGAACCCCGGG CCAGTAGCCAAGGTTCGCCATGATGACTGATCG |
|  |  | *TCGA*CGATCAGTCATCATGGCGAACCTTGGCTACTGGCCCGG GGTTCGCCATGATGACTGATCTGCAAAATGAGGG |
| **mPrP STOP** | pGF-ori-FP | *CTAG*CCTCCTTCCTCATCTTCCTGATCGTGGGATGAGGGAGGC CTTCCTGCTTGTTCCTTG |
|  |  | *AATT*CAAGGAACAAGCAGGAAGGCCTCCCTCATCCCACGATC AGGAAGATGAGGAAGGAGG |
| **mPrP 82, 121** | pGF-ori-FP | *CTAG*GCAAAAAGCGGCCAAAGCCTGGAGGGTACGCGTTGCC TCCAGGGCTTCCCTGCCCGGGAT |
|  |  | *AATT*ATCCCGGGCAGGGAAGCCCTGGAGGCAACGCGTACCCT CCAGGCTTTGGCCGCTTTTTGC |
| **mPrP +9, 39** | pGF-ori-FP | *CTAG*CTTCGCCATGATGACTGATCTGCAAAACCCGGGACATC AGTCCACATAGTCACAAAGAGG |
|  |  | *AATT*CCTCTTTGTGACTATGTGGACTGATGTCCCGGGTTTTGC AGATCAGTCATCATGGCGAAG |
| **mPrP 654, 735** | pGF-ori-FP | *CTAG*CGAAGGAGTCCCAGGCCTATTACGACGGGAGAAGATCCA GCACCCGGGGCTTTTCTCCTCCCCTCCTGTCATCCTCCTCATCG |
|  |  | *AATT*CGATGAGGAGGATGACAGGAGGGGAGGAGAAAAGCCCCGG GTGCTGGATCTTCTCCCGTCGTAATAGGCCTGGGACTCCTTCG |

*: The overhangs of the DNA linkers are marked in italics.

**Supplementary Table S3. List of the oligonucleotides used for spacer cloning into pTE4396 or pTE4398 vectors.** The matching targets, the potential cleavage positions in the ORF (when applicable) and the sequence of the oligonucleotides that form the corresponding linkers are listed

| **Target** | **Potential cleavage position** | **Oligonucleotide used** |
| --- | --- | --- |
| **1** | mouse Prion protein ORF 5^th^ bp | *AAAA*GGTTCGCCATGATGACTGATCTG |
|  |  | *AGAT*CAGATCAGTCATCATGGCGAACC |
| **2** | mouse Prion protein ORF 55^th^ bp | *AAAA*GCCGACATCAGTCCACATAGTCA |
|  |  | *AGAT*TGACTATGTGGACTGATGTCGGC |
| **3** | mouse Prion protein ORF 58^th^ bp | *AAAA*TGTCGGCCTCTGCAAAAAGCGGC |
|  |  | *AGAT*GCCGCTTTTTGCAGAGGCCGACA |
| **4** | mouse Prion protein ORF 287^th^ bp | *AAAA*CATAATCAGTGGAACAAGCCCAG |
|  |  | *AGAT*CTGGGCTTGTTCCACTGATTATG |
| **5** | mouse Shadoo protein ORF 413^th^ bp | *AAAA*TCTGCTTCTGGGCGGCACCCTTG |
|  |  | *AGAT*CAAGGGTGCCGCCCAGAAGCAGA |
| **6** | mouse Doppel protein ORF 3^th^ bp | *AAAA*TTCTTCATGGTGAATCTGCAAGG |
|  |  | *AGAT*CCTTGCAGATTCACCATGAAGAA |
| **7** | mouse Doppel protein ORF 67^th^ bp | *AAAA*CTCCACGGTCAAGGCAAGGGGCA |
|  |  | *AGAT*TGCCCCTTGCCTTGACCGTGGAG |
| **8** | mouse Doppel protein ORF 438^th^ bp | *AAAA*CCAAGCACTGCGATTTCTGGCTG |
|  |  | *AGAT*CAGCCAGAAATCGCAGTGCTTGG |
| **9** | mouse Doppel protein ORF 468^th^ bp | *AAAA*AGCGCAGCTCCCCTTTCCAGCCA |
|  |  | *AGAT*TGGCTGGAAAGGGGAGCTGCGCT |
| **10** | mouse Doppel protein ORF 519^th^ bp | *AAAA*GTTTCGTTTGGTTCATTGTGAAG |
|  |  | *AGAT*CTTCACAATGAACCAAACGAAAC |
| **11** | mouse Doppel protein ORF 537^th^ bp | *AAAA*CTTTACTTCACAATGAACCAAAC |
|  |  | *AGAT*GTTTGGTTCATTGTGAAGTAAAG |
| **12** | **-** | *AAAA*ATCAGTCATCATGGCGAACCTTG |
|  |  | *AGAT*CAAGGTTCGCCATGATGACTGAT |
| **13** | **-** | *AAAA*GCCGACATCAGTCCACATAGTCA |
|  |  | *AGAT*TGACTATGTGGACTGATGTCGGC |
| **14** | **-** | *AAAA*GCCGCTTTTTGCAGAGGCCGACA |
|  |  | *AGAT*TGTCGGCCTCTGCAAAAAGCGGC |
| **15** | **-** | *AAAA*TGATTATGGGTACCCCCTCCTTG |
|  |  | *AGAT*CAAGGAGGGGGTACCCATAATCA |
| **16** | **-** | *AAAA*TCTGCTTCTGGGCGGCACCCTTG |
|  |  | *AGAT*CAAGGGTGCCGCCCAGAAGCAGA |
| **17** | **-** | *AAAA*GATTCACCATGAAGAACCGGCTG |
|  |  | *AGAT*CAGCCGGTTCTTCATGGTGAATC |
| **18** | **-** | *AAAA*GCCCCTTGCCTTGACCGTGGAGA |
|  |  | *AGAT*TCTCCACGGTCAAGGCAAGGGGC |
| **19** | **-** | *AAAA*GCCAGAAATCGCAGTGCTTGGCG |
|  |  | *AGAT*CGCCAAGCACTGCGATTTCTGGC |
| **20** | **-** | *AAAA*CTGGAAAGGGGAGCTGCGCTTCG |
|  |  | *AGAT*CGAAGCGCAGCTCCCCTTTCCAG |

*: The overhangs of the DNA linkers are marked in italics.

**Supplementary References**

1. Cong, L., Ran, F.A., Cox, D., Lin, S., Barretto, R., Habib, N., Hsu, P.D., Wu, X., Jiang, W., Marraffini, L.A. *et al.* (2013) Multiplex genome engineering using CRISPR/Cas systems. *Science*, **339**, 819-823.

2. Ran, F.A., Cong, L., Yan, W.X., Scott, D.A., Gootenberg, J.S., Kriz, A.J., Zetsche, B., Shalem, O., Wu, X., Makarova, K.S. *et al.* (2015) In vivo genome editing using Staphylococcus aureus Cas9. *Nature*, **520**, 186-191.

3. Mashiko, D., Fujihara, Y., Satouh, Y., Miyata, H., Isotani, A. and Ikawa, M. (2013) Generation of mutant mice by pronuclear injection of circular plasmid expressing Cas9 and single guided RNA. *Sci Rep*, **3**, 3355.
