# Supplementary material for: Cpf1 nucleases demonstrate robust activity to induce DNA modification by exploiting homology directed repair pathways in mammalian cells
Source: Biol Direct. 2016 Sep 14;11:46. doi: 10.1186/s13062-016-0147-0 (PMC5024423; doi:10.1186/s13062-016-0147-0)
Supplement: Supplementary file 3 — The origin of background fluorescence of GFxFP plasmids. (DOCX 2420 kb) [file 13062_2016_147_MOESM3_ESM.docx]

**Additional file 3**

**Figure S2. The origin of background fluorescence of GFxFP plasmids**

**A.**) Comparison of cleavage efficiencies of LbCpf1 on two targets (target 6 and target 15) and of the background fluorescence found with the negative control samples (dead LbCpf1) employing GFxFP assay in N2a, HEK293T and HeLa cells. GFP positive cells are counted two days posttransfection by flow cytometry. All samples are also cotransfected with an mCherry expression vector as a transfection reference and the results are normalized to the transfection efficiencies measured by the mCherry fluorescence. Three parallel transfections were made for each case. **B.)** Number of anti-H2AX positive foci in the nucleus of untreated N2a, HEK293T and HeLa cells. Anti-H2A.X positive foci were counted in 30, randomly selected nuclei in the case of each cell line. **C.)** Representative images of anti-H2A.X staining of different cell lines. First two column: anti-H2A.X staining, third column: DAPI staining, fourth column: cells in transmitted light. Scalebar represents 50µm.
