# Supplementary material for: Cpf1 nucleases demonstrate robust activity to induce DNA modification by exploiting homology directed repair pathways in mammalian cells
Source: Biol Direct. 2016 Sep 14;11:46. doi: 10.1186/s13062-016-0147-0 (PMC5024423; doi:10.1186/s13062-016-0147-0)
Supplement: Supplementary file 4 — Schematic structure of As- (pTE4396) and LbCpf1 (pTE4398) expression plasmids with crRNA expression cassettes. (DOCX 99 kb) [file 13062_2016_147_MOESM4_ESM.docx]

**Additional file 4**

**
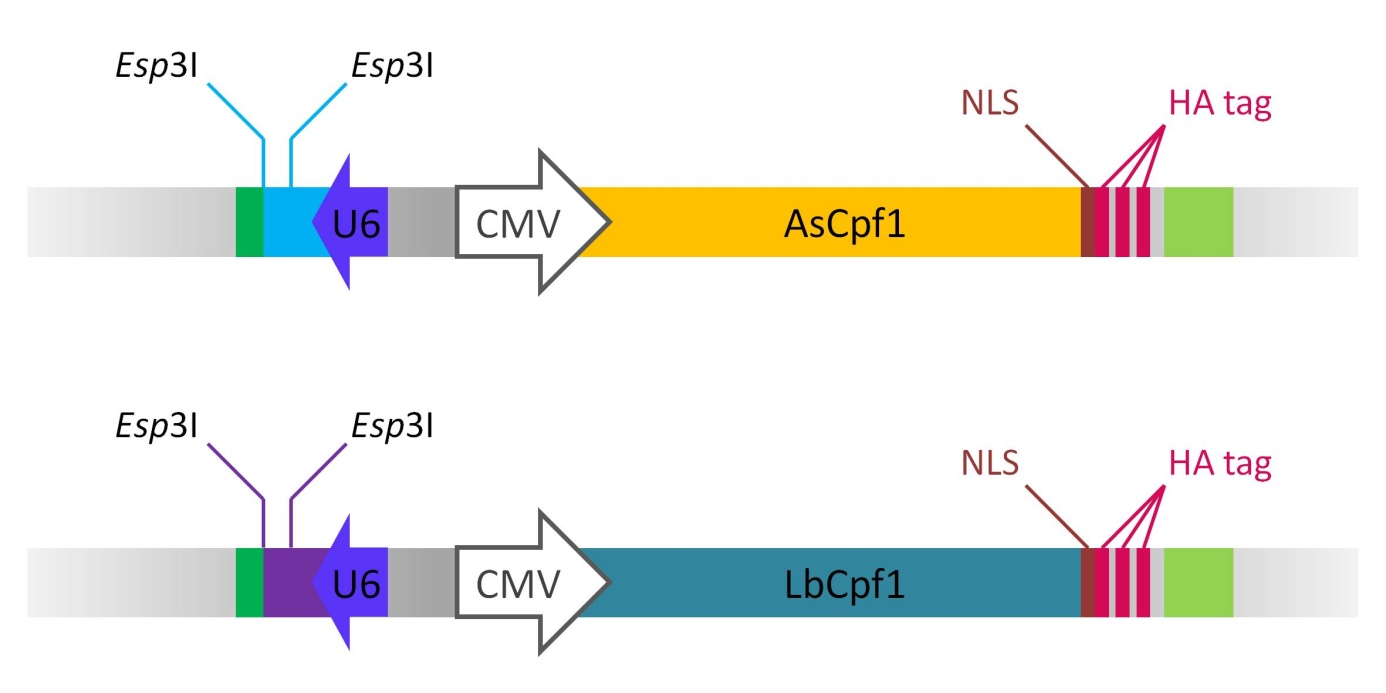
**

**Figure S3. Schematic structure of As- (pTE4396) and LbCpf1 (pTE4398) expression plasmids with crRNA expression cassettes**

green: RNA polymerase III terminator, light blue or purple: As- or LbCpf1 crRNAs (spacer can be introduced between *Esp*3I restriction enzyme sites), blue: human U6 promoter, white: CMV enhancer and promoter, orange or dark blue: As- or LbCpf1, scarlet: nuclear localization signal sequence (NLS), pink: three consecutive HA tags, light green: bGH poly(A) signal
