# Supplementary material for: Cpf1 nucleases demonstrate robust activity to induce DNA modification by exploiting homology directed repair pathways in mammalian cells
Source: Biol Direct. 2016 Sep 14;11:46. doi: 10.1186/s13062-016-0147-0 (PMC5024423; doi:10.1186/s13062-016-0147-0)
Supplement: Supplementary file 5 — LbCpf1 exert higher efficiency to induce HDR than three tested Cas9 counterparts. (DOCX 143 kb) [file 13062_2016_147_MOESM5_ESM.docx]

**Additional file 5**

**
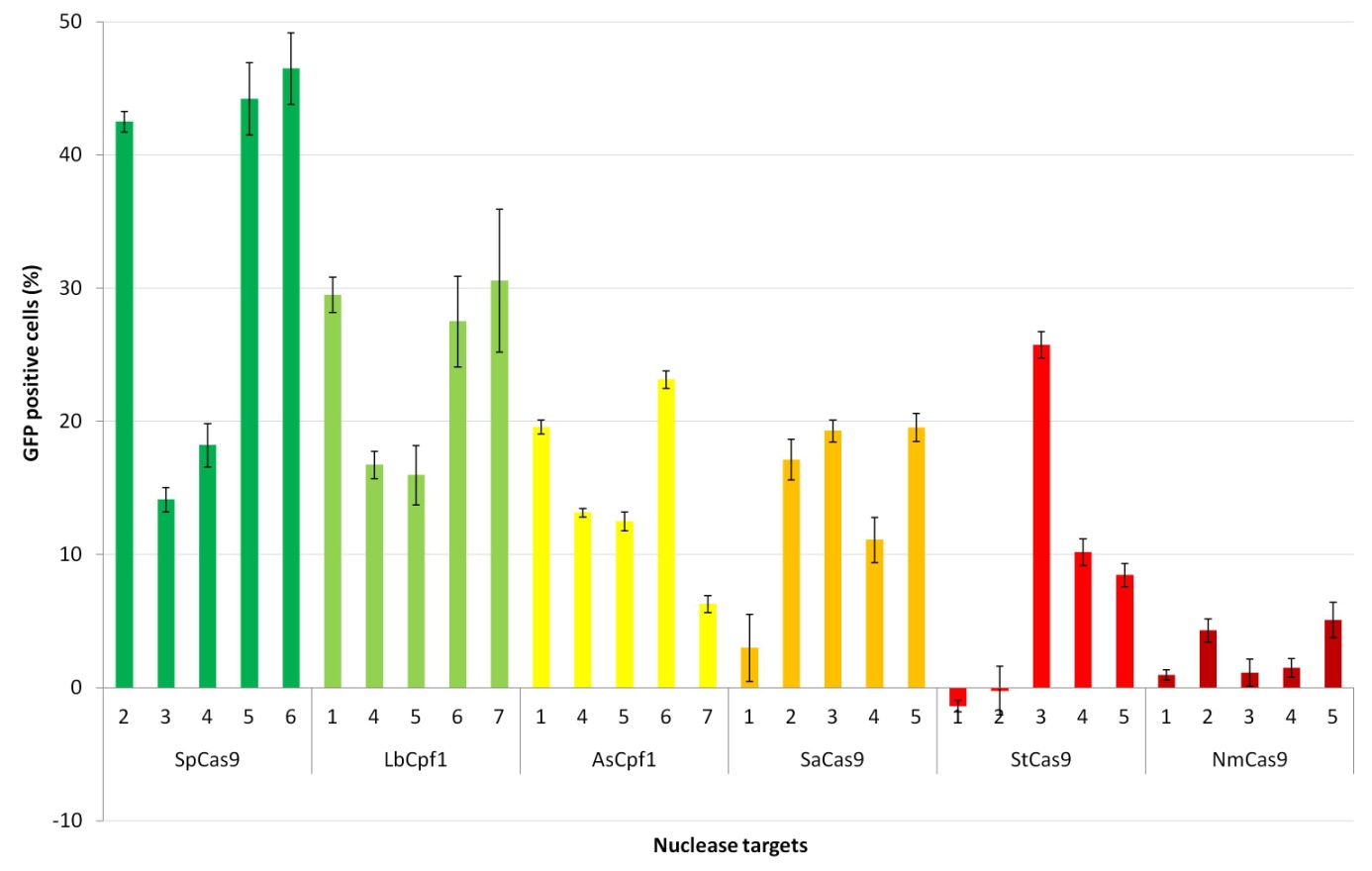
**

**Figure S4. LbCpf1 exert higher efficienct to induce HDR than three tested Cas9 counterparts**

Percentages of GFP positive cells counted above the background level, resulted by the action of various nucleases. Six nucleases were tested, each of them on five different targets using the GFxFP assay. GFP positive cells were counted two days after transfection. All samples were also transfected with a mCherry expressing vector and the results are normalized to the transfection efficiencies measured by the mCherry fluorescence. GFP fluorescence for negative controls was measured using an inactive Cas9 expressing vector (Tálas et al., unpublished results) and the obtained values are subtracted from each corresponding sample value. Three parallel transfections were made for each sample. Average HDR inducing efficiencies were considered by calculating the average of 15 (5 targets x 3 parallel samples) samples. The sequences of targets and spacers are provided in Additional file 2.
