# Supplementary material for: Cpf1 nucleases demonstrate robust activity to induce DNA modification by exploiting homology directed repair pathways in mammalian cells
Source: Biol Direct. 2016 Sep 14;11:46. doi: 10.1186/s13062-016-0147-0 (PMC5024423; doi:10.1186/s13062-016-0147-0)
Supplement: Supplementary file 6 — Homologous recombination of a GFP casette mediated by St- or SaCas9 at the PRNP locus. (DOCX 13 kb) [file 13062_2016_147_MOESM6_ESM.docx]

**Additional file 6**

**Figure S5. Homologous recombination of a GFP casette mediated by St- or SaCas9 at the PRNP locus**

Percentages of GFP fluorescent N2a cells resulted from HR mediated integration of a promoterless GFP cassette at the PRNP locus. The efficiencies of St- and SaCas9 nucleases (purple and green, respectively) to induce HR mediated integration were tested on two and five endogenous mouse PRNP targets, respectively (numbering of the samples for the corresponding targets are the same as on Supplementary Figure 4 in Additional file 5; target sequences and cleavage positions are provided in Additional file 2). Nuclease vectors and the donor plasmid were cotransfected into the cells. As negative control, an inactive SpCas9-expression vector was used to cotransfect with the donor plasmid. On the seventh day posttransfection GFP positive cells were counted. Three parallel transfections were made for each sample. Two days posttransfection all of the samples showed similar GFP positive cell counts.
