# Supplementary material for: Cpf1 nucleases demonstrate robust activity to induce DNA modification by exploiting homology directed repair pathways in mammalian cells
Source: Biol Direct. 2016 Sep 14;11:46. doi: 10.1186/s13062-016-0147-0 (PMC5024423; doi:10.1186/s13062-016-0147-0)
Supplement: Supplementary file 7 — Supplementary Materials and Methods. (DOCX 41 kb) [file 13062_2016_147_MOESM7_ESM.docx]

**Additional file 7**

**Supplementary Materials and Methods**

**Plasmid construction**

SpCas9-IRFP vector

The IRFP670 cassette was amplified from the pIRFP670-N1 plasmid [Addgene: #45457, (1)] by using Pfu polymerase (*forward primer:* ATAGCCGTCTCAGGGAGTTCCGCGTTACATAAC, *reverse primer:* ATGCTCGTCTCTCG CGCCTTAAGATACATTGATGAG). Both primers contain an *Esp*3I recognition site. The PCR product was digested with *Esp*3I and was ligated into the *Esp*3I sites of the pX330-U6-Chimeric_BB-CBh-hSpCas9 vector [Addgene: #42230, (2)]. Linkers, containing either the PrP10 or the Sp1 spacer (see Supplementary table S1 in Additional file 2 for their sequences) were inserted into the *Bbs*I sites of this vector.

pdSpCas9 and pdSpCas9-IRFP plasmids

The construction of the nuclease inactive dSpCas9 coding vector (pdSpCas9) is described in Tálas et al. (submitted manuscript). The IRFP670 cassette was inserted into the pdSpCas9 as described above for the SpCas9-IRFP plasmid to result the vector pdSpCas9-IRFP.

pGF-chl-FP plasmid

The pGF-chl-FP plasmid was constructed by ligating a chloramphenicol resistance gene into a pCAG-EGxxFP [Addgene: #50716, (3)] plasmid pre-digested by *Eco*RV. The chloramphenicol resistance gene was picked from an already existing plasmid in the laboratory by digestion (using the *Bcu*I and *Bgl*II restriction enzymes) and after Klenow-filling of the overhangs it was used for the ligation. The sequence of the chloramphenicol gene cassette is described in the Sequences section below.

pGF-ori-FP plasmid

The pGF-ori-FP plasmid was constructed from the pCAG-EGxxFP [Addgene: #50716, (3)] plasmid by the ligation of a fragment containing a pUC origin of replication (ORI) into the *Eco*RV sites of the pre-digested plasmid. The pUC ori was picked from a plasmid (pWN10019, unpublished data) by digestion with *Bgl*II and *Pag*I restriction enzymes and was used for ligation after Klenow-filling of the overhangs. The initial origin of replication was then removed from the plasmid by the *Eco*31I and *Sac*I restriction enzymes and was replaced by the *Eco*31I and *Pag*I restriction fragment of the plasmid pXXL2 (unpublished data). The *Sac*I and *Pag*I overhangs were generated by by Klenow polymerase before ligation. The sequences of the pUC origin of replication and that of the *Eco*31I and *Pag*I fragment of pXXL2 are described in the Sequences section below.

Target cloning into pGFxFP (pCAG-EGxxFP, pGF-ori-FP and pGF-chl-FP) vectors

To clone appropriate targets into these vectors, we used three different restriction enzyme pairs: *Bam*HI-*Eco*RI, *Bam*HI-*Sal*I, and *Nhe*I-*Eco*RI targeting the same multicloning site present in GFxFP vectors. The corresponding linkers containing the target sequences were ligated to these sites. The used oligonucleotides are listed in Supplementary table S2 in Additional file 2.

pcDNA3-mCherry plasmid

The ORF of mCherry was PCR amplified from a pRSETB-mCherry (unpublished results) plasmid (*forward primer:* CGCGCGGATCCACCATGGTGAGCAAGGGCGAGGAG, *reverse primer:* CGCCTGCGGCCGCTCACTTGTACAGCTCGTCCATG) using Pfu polymerase and was inserted between the *Bam*HI and *Not*I restriction enzyme sites of a pcDNA3 vector (Invitrogen) to create the plasmid pcDNA3-mCherry. The sequence of the mCherry is described in the Sequences section below.

pAsCpf1-DNMT1.3 and pLbCpf1-DNMT1.3 vectors

The human U6 promoter and the DNMT1.3 targeting crRNA of either As- or LbCpf1 were inserted between the *Bcu*I and *Mun*I restriction enzyme sites of the corresponding expression plasmids for the nucleases, pY010 and pY016 [Addgene: #69982 and #69988 (4)], respectively, to create the pAsCpf1-DNMT1.3 or pLbCpf1-DNMT1.3 vectors. The human U6 promoter was generated from a px330 vector [Addgene: #42230, (2)] by PCR amplification (*forward primer*: TTTTTTACTAGTGAGGGCCT ATTTCCCAT; *reverse primer*: GGGGGGCAATTGGCTCTTCGGTGTTTCGTCCTTTCC) using Q5 polymerase. The crRNAs were generated by hybridization of the following oligonucleotides: As#1: CACCGTAATTTCTACTCTTGTAGATCTGATGGTCCATGTCTGTTACTCTTTTTT; As#2: AATTAAAAAAGAGTAACAGACATGGACCATCAGATCTACAAGAGTAGAAATTACG for AsCpf1 and Lb#1: CACCGTAATTTCTACTAAGTGTAGATCTGATGGTCCATGTCTGTTACTCTTTTTT; Lb#2: AATTAAAAAAGAGTAACAGACATGGACCATCAGATCTACACTTAGTAGAAATTACG) for LbCpf1. Each annealed oligonucleotide was ligated to the PCR fragment pre-digested by *Lgu*I and *Mun*I.

pAsCpf1-crRNA (pTE4396) and pLbCpf1-crRNA (pTE4398) plasmids

pAsCpf1-crRNA (pTE4396) and pLbCpf1-crRNA(pTE4398) were constructed as described for pAsCpf1-DNMT1.3 vector, using oligonucleotides #1: AATTAAAAAAGGAGACGGGATCCCGTCTCCATCTACAAGAGTAGAAATTACG; #2: CACCGTAATTTCTACTCTTGTAGATGGAGACGGGATCCCGTCTCCTTTTTT for AsCpf1, and #1: AATTAAAAAAGGAGACGGGATCCCGTCTCCATCTACACTTAGTAGAAATTAC; #2: CACCTAATTTCTACTAAGTGTAGATGGAGACGGGATCCCGTCTCCTTTTTT for LbCpf1. The spacer sequence in the resulted plasmids can be replaced by using *Esp*3I restriction enzyme.

Cpf1 spacer cloning

To modify the spacer sequence of As- and LbCpf1 crRNAs a one-pot digestion-ligation (5) protocol was developed. The synthetic DNA oligonucleotides listed in Supplementary table S3 in Additional file 2 were hybridized and samples were cooled from 95 to 4 °C in one hour. The annealed oligonucleotides (2.5 µM) with 50 ng acceptor vector (pTE4396 or pTE4398), 3 units of *Esp*3I restriction enzyme, and 1.5 units of T4 DNA ligase were mixed in Tango buffer (Thermo Fisher Scientific) containing 1 mM DTT (Sigma-Aldrich) and 500 µM ATP. The mixture was kept at 37 °C for one hour before transforming into chemically competent, stable *E. coli* cells (NEB). Two single colonies, formed after culturing on agar plate, were tested by restriction enzyme digestion and appropriate clones were sent for sequencing.

pTE4254-StCas9 plasmid

First, the unique Esp3I restriction enzyme site was deleted in the pM-ST1cas [Addgene #48669, (6)] vector, by using a small linker between *Sac*I and *Bam*HI sites, resulting the phStCas9 vector. The human U6 promoter was amplified from the pSimpleII-U6-tracr-U6-BsmBI-NLS-NmCas9-HA-NLS(s) plasmid [Addgene: #47868, (7)] using Q5 polymerase (forward primer: GGGGGGCAATTGCGTACGAAAAAAACGAAATGAGAAAGG, reverse primer: GGGGGGGGCGCGCCAACTAGCATATCGATATGGATTCG, containing an *Esp*3I enzyme site) and was digested by *Sgs*I and *Mun*I restriction enzymes before it was inserted into the *Mun*I and *Mlu*I sites of the phStCas9 vector resulting phStCas9-U6. The StCas9 gRNA was introduced into this plasmid by ligating three small DNA linkers (#1: AATTGCTTAAGAAAAAAACACCCTGCCATAAAATGACAGGGTGTTGATTT, #2: CATGCCGAAATCAACACCCTGTCATTTTATGGCAGGGTGTTTTTTTCTTAAGC, #3: CGGCATGAAGCCTTATCTTTGTAGCTTCTGCAAGATTTAAGTAACTGTG, #4: AAACTTACACAGTTACTTAAATCTTGCAGAAGCTACAAAGATAAGGCTT, #5: TAAGTTTCGTTGTACAGTTACTTAAATCTTGAGAGTACAAAAACGGAGACGGGTACCCGTCTCC, #6: CACCGGAGACGGGTACCCGTCTCCGTTTTTGTACTCTCAAGATTTAAGTAACTGTACAACG) into the *Mun*I and *Esp*3I sites.

pKS7107-NmCas9 plasmid

# The crRNA of NmCas9 was introduced between the *Esp*3I restriction enzyme sites of pSimpleII-U6-tracr-U6-BsmBI-NLS-NmCas9-HA-NLS [Addgene #47868, (7)] using a linker (#1: AAAACGAAATGAGAAAGGGAGCTACAACGGAGACGGGATCCCGTCTCC, #2: CACCGGAGACGGGATCCCGTCTCCGTTGTAGCTCCCTTTCTCATTTCG).

Cas9 spacer cloning

The one-pot digestion-ligation protocol of Cpf1 spacer cloning was followed except that we used *Eco*31I restriction enzyme for SaCas9 gRNA spacer cloning and *Esp*3I for StCas9 gRNA and NmCas9 crRNA spacer cloning. The sequences of the spacers used are listed in Supplementary table S1 in Additional file 2).

dead LbCpf1

Inactive LbCpf1 was generated by mutating the D832 to alanine. The LbCpf1 vector [pY016, Addgene: #69988 (4)] was amplified in two fragments with two, overlapping primer pairs (#1: GCGGGACTCTGGGGTTCG, #2: CGCGATGCCGATCACATAGGGGT, #3: ATGTGATCGGCATC GCGAGGGGCGAGCGCAATCTG, #4: AACCCCAGAGTCCCGCTCAGAAGAACTC). One of the primer pairs contain the D832A mutation. The PCR fragments were transformed into E. coli competent cells.

**Sequences**

chloramphenicol resistance gene cassette

actagtgcttggattctcaccaataaaaaacgcccggcggcaaccgagcgttctgaacaaatccagatggagttctgaggtcattactggatctatcaacaggagtccaagcgagctcgatatcaaattacgccccgccctgccactcatcgcagtactgttgtaattcattaagcattctgccgacatggaagccatcacaaacggcatgatgaacctgaatcgccagcggcatcagcaccttgtcgccttgcgtataatatttgcccatggtgaaaacgggggcgaagaagttgtccatattggccacgtttaaatcaaaactggtgaaactcacccagggattggctgagacgaaaaacatattctcaataaaccctttagggaaataggccaggttttcaccgtaacacgccacatcttgcgaatatatgtgtagaaactgccggaaatcgtcgtggtattcactccagagcgatgaaaacgtttcagtttgctcatggaaaacggtgtaacaagggtgaacactatcccatatcaccagctcaccgtctttcattgccatacgaaattccggatgagcattcatcaggcgggcaagaatgtgaataaaggccggataaaacttgtgcttatttttctttacggtctttaaaaaggccgtaatatccagctgaacggtctggttataggtacattgagcaactgactgaaatgcctcaaaatgttctttacgatgccattgggatatatcaacggtggtatatccagtgatttttttctccattttagcttccttagctcctgaaaatctcgataactcaaaaaatacgcccggtagtgatcttatttcattatggtgaaagttggaacctcttacgtgccgatcaagatct

pUC origin of replication

agatctcatgtgagcaaaaggccagcaaaaggccaggaaccgtaaaaaggccgcgttgctggcgtttttccataggctccgcccccctgacgagcatcacaaaaatcgacgctcaagtcagaggtggcgaaacccgacaggactataaagataccaggcgtttccccctggaagctccctcgtgcgctctcctgttccgaccctgccgcttaccggatacctgtccgcctttctcccttcgggaagcgtggcgctttctcatagctcacgctgtaggtatctcagttcggtgtaggtcgttcgctccaagctgggctgtgtgcacgaaccccccgttcagcccgaccgctgcgccttatccggtaactatcgtcttgagtccaacccggtaagacacgacttatcgccactggcagcagccactggtaacaggattagcagagcgaggtatgtaggcggtgctacagagttcttgaagtggtggcctaactacggctacactagaagaacagtatttggtatctgcgctctgctgaagccagttaccttcggaaaaagagttggtagctcttgatccggcaaacaaaccaccgctggtagcggtggtttttttgtttgcaagcagcagattacgcgcagaaaaaaaggatctcaagaagatcctttgatcttttctacggggtctgacgctcagtggaacgaaaactcacgttaagggattttggtcatga

pXXL2 *Eco*31I-*Pag*I fragment

tcatgagattatcaaaaaggatcttcacctagatccttttaaattaaaaatgaagttttaaatcaatctaaagtatatatgagtaaacttggtctgacagttaccaatgcttaatcagtgaggcacctatctcagcgatctgtctatttcgttcatccatagttgcctgactccccgtcgtgtagataactacgatacgggagggcttaccatctggccccagtgctgcaatgataccgcgagacc

mCherry

ccatggtgagcaagggcgaggaggataacatggccatcatcaaggagttcatgcgcttcaaggtgcacatggagggctccgtgaacggccacgagttcgagatcgagggcgagggcgagggccgcccctacgagggcacccagaccgccaagctgaaggtgaccaagggtggccccctgcccttcgcctgggacatcctgtcccctcagttcatgtacggctccaaggcctacgtgaagcaccccgccgacatccccgactacttgaagctgtccttccccgagggcttcaagtgggagcgcgtgatgaacttcgaggacggcggcgtggtgaccgtgacccaggactcctccctgcaggacggcgagttcatctacaaggtgaagctgcgcggcaccaacttcccctccgacggccccgtaatgcagaagaagaccatgggctgggaggcctcctccgagcggatgtaccccgaggacggcgccctgaagggcgagatcaagcagaggctgaagctgaaggacggcggccactacgacgctgaggtcaagaccacctacaaggccaagaagcccgtgcagctgcccggcgcctacaacgtcaacatcaagttggacatcacctcccacaacgaggactacaccatcgtggaacagtacgaacgcgccgagggccgccactccaccggcggcatggacgagctgtacaagtaag

**Immuncytochemistry**

Cells (N2a, HeLa, HEK293T) were cultured on Labtek II 8 well chambers (Labtek), seeded at a density of 5*10^4 cells/well. Two days after seeding, cells were fixed using 4% paraformaldehyde in PBS for 10 minutes. Blocking and permeabilizing mixture contains Triton-X 100 and BSA. Primary antibody (anti-H2A.X, BioLegend) was used in 1:200 dilution, secondary antibody was used in 1:200 dilution (DyLight 488 anti-mouse, Pierce). Nuclei were stained by DAPI (1:10,000, Sigma).

**Supplementary References**

1. Shcherbakova, D.M. and Verkhusha, V.V. (2013) Near-infrared fluorescent proteins for multicolor in vivo imaging. *Nat Methods*, **10**, 751-754.

2. Cong, L., Ran, F.A., Cox, D., Lin, S., Barretto, R., Habib, N., Hsu, P.D., Wu, X., Jiang, W., Marraffini, L.A. *et al.* (2013) Multiplex genome engineering using CRISPR/Cas systems. *Science*, **339**, 819-823.

3. Mashiko, D., Fujihara, Y., Satouh, Y., Miyata, H., Isotani, A. and Ikawa, M. (2013) Generation of mutant mice by pronuclear injection of circular plasmid expressing Cas9 and single guided RNA. *Sci Rep*, **3**, 3355.

4. Zetsche, B., Gootenberg, J.S., Abudayyeh, O.O., Slaymaker, I.M., Makarova, K.S., Essletzbichler, P., Volz, S.E., Joung, J., van der Oost, J., Regev, A. *et al.* (2015) Cpf1 is a single RNA-guided endonuclease of a class 2 CRISPR-Cas system. *Cell*, **163**, 759-771.

5. Engler, C., Kandzia, R. and Marillonnet, S. (2008) A one pot, one step, precision cloning method with high throughput capability. *PLoS One*, **3**, e3647.

6. Esvelt, K.M., Mali, P., Braff, J.L., Moosburner, M., Yaung, S.J. and Church, G.M. (2013) Orthogonal Cas9 proteins for RNA-guided gene regulation and editing. *Nat Methods*, **10**, 1116-1121.

7. Hou, Z., Zhang, Y., Propson, N.E., Howden, S.E., Chu, L.F., Sontheimer, E.J. and Thomson, J.A. (2013) Efficient genome engineering in human pluripotent stem cells using Cas9 from Neisseria meningitidis. *Proc Natl Acad Sci U S A*, **110**, 15644-15649.
